# Inappropriate Use of Medication by Elderly, Polymedicated, or Multipathological Patients with Chronic Diseases

**DOI:** 10.3390/ijerph15020310

**Published:** 2018-02-10

**Authors:** Virtudes Pérez-Jover, José J. Mira, Concepción Carratala-Munuera, Vicente F. Gil-Guillen, Josep Basora, Adriana López-Pineda, Domingo Orozco-Beltrán

**Affiliations:** 1 Health Psychology Department, Miguel Hernandez University, 03202 Elche, Alicante, Spain; v.perez@umh.es (V.P.-J.); jose.mira@umh.es (J.J.M.); 2 Sant Joan-Alacant Health District, Conselleria Sanitat, 03202 Elche, Alicante, Spain; jbasora@semfyc.es; 3 Clinical Medicine Department, Miguel Hernandez University, 03550 San Juan de Alicante, Alicante, Spain; vte.gil@gmail.com (V.F.G.-G.); adriannalp@hotmail.com (A.L.-P.); dorozcobeltran@gmail.com (D.O.-B.)

**Keywords:** chronic disease, physicians, primary care, patient medication knowledge, patient safety, medication errors, polypharmacy

## Abstract

The growth of the aging population leads to the increase of chronic diseases, of the burden of multimorbility, and of the complexity polypharmacy. The prevalence of medication errors rises in patients with polypharmacy in primary care, and this is a major concern to healthcare systems. This study reviews the published literature on the inappropriate use of medicines in order to articulate recommendations on how to reduce it in chronic patients, particularly in those who are elderly, polymedicated, or multipathological. A systematic review of articles published from January 2000 to October 2015 was performed using MEDLINE, EMBASE, PsychInfo, Scopus, The Cochrane Library, and Index Medicus databases. We selected 80 studies in order to analyse the content that addressed the question under consideration. Our literature review found that half of patients know what their prescribed treatment is; that most of elderly people take five or more medications a day; that in elderly, polymedicated people, the probability of a medication error occurring is higher; that new tools have been recently developed to reduce errors; that elderly patients can understand written information but the presentation and format is an important factor; and that a high percentage of patients have remaining doubts after their visit. Thus, strategies based on the evidence should be applied in order to reduce medication errors.

## 1. Introduction

The older, multimorbid population with chronic conditions is expanding globally, and this inevitably means a growth in complex polypharmacy and associated problems [1]. The frequency of medication errors is high in patients with polypharmacy in primary care [2]. Medication errors are a major concern to healthcare systems [3] because they can reduce the therapeutic effectiveness and represent an incremental cost [4]. It is common to analyse the role of professionals in medication errors. However, in the case of primary care, previous studies have also underlined the role that patients and their caregivers play in these mistakes [4,5,6]. Patient medication errors usually have minor consequences or none at all but, in some cases, they have serious health consequences, including hospitalization and death [4]. 

The quality of communication between the clinician and the patient [7] has been highlighted as one of the key factors in explaining the inappropriate use of medicines by patients, but it is not the only one [4,5]. For this reason, different strategies have been developed to help patients manage their medication, avoid missed doses [6,7], and avoid the most common blunders regarding treatment [8], especially in patients who are elderly, dependent, polymedicated, and multipathological [2,9,10,11]. Any measure that can increase patient safety in primary care will have an immediate multiplier effect in a large number of patients, making this type of intervention very important [3,12]. The aim of this study is to review the published literature on the inappropriate use of medicines and to articulate recommendations on how to reduce it in chronic patients, particularly those who are elderly, polymedicated, or multipathological.

## 2. Materials and Methods

### 2.1. Search Strategy and Inclusion Criteria

We carried out a systematic review of articles published from January 2000 to October 2015 and indexed in MEDLINE, EMBASE, PsychInfo, Scopus, The Cochrane Library, and Index Medicus. We followed the guidelines of the Preferred Reporting Items for Systematic Reviews and Meta-Analyses (PRISMA) and used Medical Subject Headings (MeSH), controlled vocabulary, and other key or free text words in our search strategies. The search algorithm used for MEDLINE (via PubMed) was “Attitude of Health Personnel (MeSH) AND Medical Errors” (MeSH) OR “Patient Harm” (MeSH) AND Patient Education as Topic/organization & administration (MeSH) OR (Patient Medication Knowledge/organization & administration* (MeSH), OR Medication Adherence (MeSH), AND (Polypharmacy, wrong doses, misuses medication) OR (Prescription Drug Misuse (MeSH) OR self-medication OR over-the-counter medication), AND (leaflets legibility OR patient information leaflets). The algorithm was adapted for the rest of the databases according to their own specifications. We limited results to documents published in French, English, and Spanish.

In addition to the database search, handsearches of grey literature were performed by topic experts. The inclusion criteria were citable and empirical studies, as defined by the Web of Science (original articles and reviews), on the inappropriate use of medicines and medication errors of patients at home (dosage, confusion about which drug to take, storage error). Exclusion criteria were studies on lack of therapeutic adherence, overdose due to intentional suicide attempt, or non-citable papers such as editorials or letters to the editor.

### 2.2. Article Screening

Two reviewers independently examined the title, abstract, and/or full text of the eligible articles identified by the search engines of each database to determine their relevance. Any discrepancies were resolved by consulting a third investigator. This process resulted, at times, in the identification of other pertinent publications.

### 2.3. Data Extraction

Two researchers organised the information collected during the literature review around frequently asked questions (FAQs). These questions were defined after the review by consensus of the research team, considering topics usually analysed in the reviewed studies. The criteria to choose these topics considered their impact in assuring patient safety when elderly patients are using medication at home. These FAQs responded with possible ways to foster patient safety for people with chronic disease through the appropriate use of medicines, with a special focus on the elderly.

For the narrative analysis, the information was organised around eight questions: (1) How many elderly patients with chronic diseases take five or more medications daily? (2) What are the most common medication errors in elderly patients with chronic diseases? (3) To what extent do elderly patients taking five or more daily medications understand what they are taking and why? (4) What level of treatment adherence do elderly patients with chronic diseases have? (5) What tricks do patients use to remember to take their medication? (6) Are pill organisers useful? (7) Is it more difficult for elderly patients to understand written information, for example the information leaflets for medicines? (8) How many patients have doubts or want to ask their doctor something about their medication?

## 3. Results

**Figure 1:**
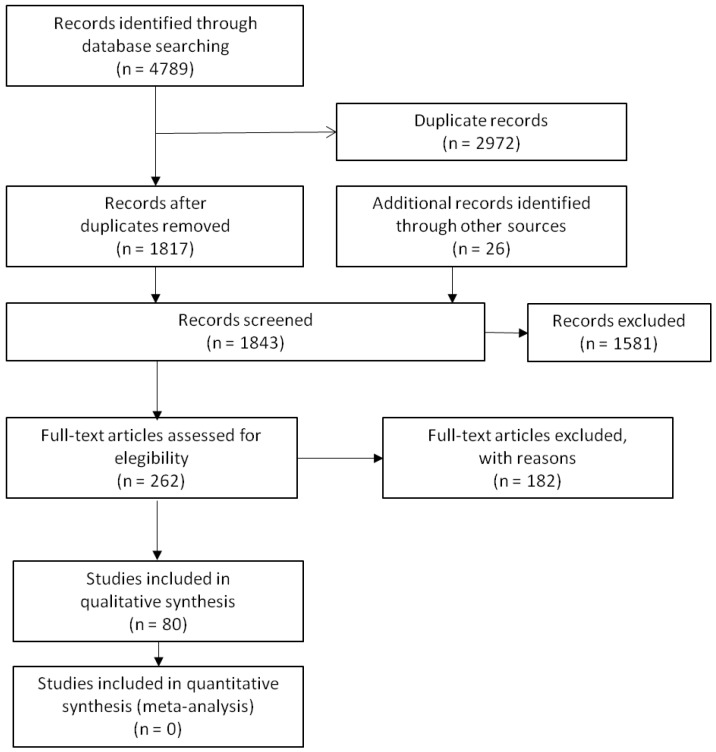
PRISMA flow diagram.

We identified a total of 4789 records in the databases consulted. Of these, 2972 were duplicates of original articles. We excluded 1581 records because they did not deal with our research questions. We examined 236 full-text articles, which led to the manual identification of an additional 26 articles that were not captured by the search engines. Finally, we selected 80 studies in order to analyse the content that addressed the questions under consideration (Figure 1). Table 1 describes the country, main topic, and the evidence level of the research reviewed using the Scottish Intercollegiate Guidelines Network (SIGN) [13].

### 3.1. Frequently Asked Questions (FAQs)

1. How many elderly patients with chronic diseases take five or more medications daily?

Worldwide, more than 46% of people aged 60 years and over have disabilities and more than 250 million older people experience moderate to severe disability [14]. Polypharmacy in the elderly is increasing and has been identified as a medication safety issue. The risk of an adverse event increases exponentially for five medications [2,15].

In Australia, people over 65 years of age have an average of 2.8 chronic illnesses, a figure that rises to 3.23 in those over 75. Among these patients, 94% take five or more medications a day, and more than half are under the care of several physicians at once [11]. This group was responsible for more than 30% of total medicine use in Spain and 73% of pharmaceutical expenditure, and they span over 65% of the total therapeutic groups [16]. In Austria, patients over 75 years of age took an average of 9.1 medications per day, and the risk of medication error is higher in this group [2]. Lizano-Díez [17] analysed medication use in all polymedicated patients attending primary care centres in Catalonia, finding that the plurality of prescriptions (40% of the total invoiced) pertained to patients aged 75 to 84. These figures show that physicians might remember that polypharmacy is the best predictor of potential medication interactions [15].

When attending a patient with this profile, the primary care physician could consider the probability that the patient is being seen by an average of more than two doctors, and each will have prescribed treatment [5]. When asked about what medications they are taking, many patients will respond that they are only taking the medicines prescribed by that physician, so it may be necessary to remind them to list all of the medications they are taking, including herbal or home remedies, in order to avoid interactions [18].

2. What are the most common medication errors in elderly patients with chronic diseases?

Among elderly, polymedicated people, the probability of medication errors at home is high [5,19,20,21]. In Spain, around 38.7% of patients made a mistake with their medication in the previous year [16,22]. Studies conducted in USA or European countries yielded similar figures [4]. The most common errors reported by elderly, polymedicated, and multipathological people were skipping a dose or not taking it regularly or at the prescribed frequency (reported by 50% of respondents), not remembering how the doctor had said to take the medication (42.9%), and confusing different medications and not taking the right one (24.9%). Less frequent errors included taking a higher dose than what was prescribed (7.9%) and mixing medications that patients were told not to take together (5%). Among the patients who committed an error, 5% had to go to hospital and 3.9% required a new treatment.

Furthermore, if we look at studies that use the system of classifying medication errors proposed by Otero, Codina, Tamés, and Pérez [23], we observe that although the rate is different, skipping medication is one of the mistakes repeated most often. Thus, Fernández Lison et al. [22] found that the most common errors were related to the wrong frequency of administration (occurring in 29% of the cases studied), therapeutic duplication (27%), and drug omission (24%). For Pérula de Torres et al. [24], the most common errors had to do with taking a smaller dose than the correct one (14.7%), drug omission (13.8%), drug duplication (6.9%), and drug deterioration (6.9%).

In USA, Australia, or Spain studies have suggested that when the number of prescriptions rises (seven or more drugs), it is more common to confuse the medications (*p* = 0.002) [18] given that, as the number of medications that patients take increases, their understanding of the correct dosage for each decreases [25,26]. This is an important consideration as the prevalence of polymedication in patients aged 65 and older is about 50%, and these patients (for example, in Spain) are taking an average of nine drugs each [9,27].

3. To what extent do elderly patients taking five or more daily medications understand what they are taking and why?

Polymedicated patients have a limited knowledge of prescribed medication [25]. If patients were asked what they know about the treatment they were taking, the rate of correct response was about 52% considering all of the medications prescribed [28]. They were most knowledgeable about which medicines they were taking (75.8% of patients responded correctly to this item), what the medicines were for, and how to take them (67.8%). Patients were least knowledgeable about the precautions they should take when using their medications (with only a 3.9% rate of correct responses).

Akici et al. [29] reported that Turkish patients, particularly who are poorly educated, males, and who received a first prescription, know little about their prescribed drugs. Similar results have been observed in other countries [7]. However, patients who are taking the same medications for a long time should be considered to remember the name of the medication, what they are taking it for, and how they should take it because this is usually the information the physician repeats at each consultation. In addition, elderly patients are worried about tools and tricks to order all the medicines they take, especially as they take more medicines. When adjusting the number of correct responses for the number of prescriptions, Mira JJ et al. [28] observed that people with five to six prescriptions were more likely to forget which medicines they were taking than people with seven or more. Specifically, in answer to the question, “What drugs are you taking?” patients with five to six prescriptions answered correctly 68.3% of the time, whereas the rate of correct response was 76.9% in those with seven to eight prescriptions, 79.8% in those with nine to ten prescriptions, and 80.4% in those with eleven or more prescriptions. Meanwhile, in answering the question, “What precautions are you taking in the use of the drug?” this is usually not included in the physician’s instructions, and patients with five to six prescriptions answered correctly 5.9% of the time, whereas the rate of correct response was 3.5% in those with seven to eight prescriptions, 2.5% in those with nine to ten prescriptions, and 3.2% in those with eleven or more prescriptions. Men and women answered correctly at a comparable rate; interestingly, the number of years in treatment did not increase the likelihood of responding correctly.

4. What level of treatment adherence do elderly patients with chronic diseases have?

Adherence affects patient outcomes, especially for chronic diseases, due to the negative consequences associated with non-adherence (i.e., larger number of hospitalisations and increase in healthcare costs) [19,30]. The rates of non-adherence around the world range from 7.1 to 66.2% in the literature [31,32,33] and exceed 50% among the polymedicated elderly [34]. Evidence indicated that adherence poses significant challenges, and the first year of treatment appears to be crucial, with 50% of dropouts occurring in the first six months of treatment [35].

The complexity of the therapeutic regimen entailed in polymedication, the confusion surrounding generics, the lack of understanding about pharmacotherapeutic regimens, and the number of prescribers, along with factors inherent to the patients themselves (e.g. memory loss or negative perceptions towards some drugs), were the most frequently cited causes of non-adherence everywhere [19,25,30,36,37,38,39,40].

Considering that the rates of non-adherence can be largely attributed to involuntary forgetfulness, studies from Sweden or Canada have shown it is essential that healthcare professionals ask patients what they do when they forget to take their medication, in order to ensure that they know the correct and safe course of action in each case [6,41]. It is important that clinicians are able to advise their patients correctly on what to do in case of the most common doubt: whether or not they have taken their medication [42].

5. What tricks do patients use to remember to take their medication?

To improve adherence and reduce errors, patients can use tricks that other patients from several countries have found to be useful [43,44]. In Mira et al. [28], a total of 155 (58.5%) Spanish polymedicated, multipathological patients aged 65 and over reported using memory tricks or other systems of reinforcement (for example, associating mealtimes with medication, recording the dosage on the box, storing the medication in a special place, leaving it in plain sight in the living room, or putting the prescription sheet on the refrigerator) and 122 (46%) used pill organisers to adequately manage medication in their home. 

Recent studies from Asia, North America, and Europe have developed and assessed the use of apps to reduce medical errors at home [4]. The use of apps opens new possibilities in helping patients take their medication correctly [45,46]. The introduction of QR codes (for ‘quick response’) on drug containers, for example, allows patients to receive oral instructions when they scan the code with their smartphone [47].

6. Are pill organisers useful?

Although patients use several memory tricks the most studied tool to avoid forgetfulness is pill organisers. This tool, also known as personalised dosage systems (PDS), can help patients both remember to take their medication and remind them whether or not they have. The use of the PDS as a strategy to improve adherence in elderly, polymedicated patients is widely recommended [4,10,31,48,49,50]. Existing studies show that PDS do seem to increase adherence, although results on the strength of effect vary. In a randomised controlled trial in 220 polymedicated patients aged 70 or older, Morales et al. [31] found that adherence improved in patients who had been given a PDS. Although the differences were not statistically significant, adherence doubled after two months of intervention (i.e., from 6% at the baseline to 12% at the trial end point). Serra-Prat, Bartolomé, Fité and Agustí [48] also observed improvement when measuring adherence by tallying unused pills in an elderly, polymedicated population; they observed an adherence rate of 73.5% in the control group, compared to 98.9% in the PDS group (*p* = 0.001). In their study of polymedicated patients over 60 with hypertension and/or dislipidaemia, Llaves, Segura, García-Jiménez and Baena [49] reported a significant 23% increase in treatment adherence, from 40% at baseline to 63% eight weeks after beginning use of the device (*p* < 0.02).

More recently, with the development of new technologies and the expansion of the use of smartphones around the world, different interventions for adherence have been designed for use with mobile apps [51,52,53,54]. Research on these tools has found them to be effective and to contribute to greater patient autonomy [55,56]. A recent intervention study to determine the usefulness of a virtual pill organiser for increasing treatment adherence in 99 polymedicated, multipathological participants aged 65 and over found that patients in the intervention group reported a higher level of treatment adherence (measured using the Morrisky-Green Test) and a lower frequency of skipped doses by the study’s end [50]. The increase in adherence in this case was 28.3%, and the number of memory lapses that participants reported related to medication fell by 28.12%.

7. Is it more difficult for elderly patients to understand written information, for example the information leaflets for medicines?

Both oral and written information are crucial in involving patients in decisions regarding their health [57,58], in fostering the safe use of medication [18], in achieving better treatment adherence [59], and in increasing their satisfaction with the care received [60]. As an important share of information transmitted orally may be forgotten [61,62], written information should be provided to reiterate and reinforce the message [63]. Studies focusing on information for patients, in the form of leaflets or healthcare websites, have demonstrated the differences in what kind of information doctors want to give and what kind patients want to receive [64,65,66]. Researchers from several countries have also found that the presentation and format is an important factor in ensuring that the information gets to the target audience [57,67] and that leaflets and websites that complement oral explanations do, in fact, help patients to be more knowledgeable about their health and addressing issues around it [68].

A Spanish study that compared information provided in medication leaflets versus other sources found that the information in leaflets was more comprehensive and understandable. None of the sources analysed had much information about common patient errors when using the medication [69]. Another study evaluated blogs and wikis created by patient associations and the pharmaceutical industry, also finding that these media offered only incomplete information on medicines [70].

The quality of written information for patients has been analysed from different angles, although the main focus has been on assessing the readability of the texts. This aspect is measured by the Flesch or LEGIN indexes [59,61] or through scales like DISCERN [71]. Depending on the study, readability as measured by the Flesch index is acceptable [72] or low [73,74]. In the specific case of medication leaflets, investigators have underlined that there is little blank space and that the type is too small, making it difficult read [75,76]. Barrio-Cantalejo and Simón-Lorda [77] analysed the readability of 326 health education leaflets according to Flesch readability, sentence complexity, and LEGIN indexes, reporting that 40% of the analysed texts failed to fulfil at least one readability criterion (i.e., excessively long words and sentences) and 56% used small print that was unsuitable for older readers. March et al. [78] reached the same conclusions in their qualitative analysis of leaflets based on patient and professional perspective; these authors highlighted that the overly technical language and small print made the media difficult to read.

Although there is a tendency to believe that elderly patients have more difficulties in understanding written information, this does not appear to be the case. Rather, patients’ education level and familiarity with the topic are more important than their age [79].

8. How many patients have doubts or want to ask their doctor something about their medication?

It is not surprising that despite expressing their satisfaction with the information provided by their physician, patients from several countries also report that they do not have all of the information they would like to have [80,81,82,83] or that they have doubts and concerns that they never raise with their provider [84]. Patients and professionals usually make different value judgements with regards to what information they consider relevant in the course of treatment. For example, patients would prefer having more information on the precautions they should take in order to use their medication correctly [77].

Barca et al. [85] found that 22.7% (95% confidence interval (CI) 16.41–30.36) of the Spanish patients they interviewed reported leaving the consultation without asking a question they had about their treatment. Of these patients, 29.4% did not ask for any information at all. Furthermore, 18.67% (95% CI 12.96–26.02) said they did not understand all of the information they had been given, and 39.28% of these, likewise, did not ask their doctor any questions.

The duration of the consultation seems to be a decisive factor in determining whether patients have remaining doubts after their visit [86]. Of 8953 adults and 6329 paediatric patients attended in primary care, 19.6% and 12.1%, respectively, stated that they did not have enough time to speak to their doctor. When the consultation lasted less than 10 min, patients were more likely to report not receiving sufficient information (*p* < 0.001) and to require an additional consultation due to a problem with their treatment (*p* < 0.0001).

### 3.2. Recommendations

The older population is increasing and most of elderly people are polymedicated. Available evidence shows that polymedicated patients have a limited knowledge of prescribed medication [25] and that the probability of a medication error occurring is high [2]. Besides increasing adverse events and reducing the therapeutic effectiveness, medication errors are a burden for the health systems. Thus, in order to reduce them strategies based on the evidence should be applied. 

Through this review, the most important issues that lead to the inappropriate use of medicines can be identified. This information can be used to suggest recommendations to healthcare providers. In order to avoid the major concerns, physicians should ask patients if they have doubts during the visit, preventing the most common errors and equipping them with strategies to avoid them. It is important that the physician knows who is preparing and administering the medicines, whether it is the patient themselves or a family member or other caregiver, as the explanations given in the consultation are not always repeated correctly to the person responsible for administering the drugs at home.

In addition, simply organised, written information can help polymedicated patients to understand their treatment regimen. The physician should take the precaution of warning patients of the risks involved in taking the medication clearly and directly (for example, not mixing it with another drug, taking it with food, or any indication on storage instructions when applicable). Physicians should also ensure that this information is transmitted to the person responsible for administering the medication if it is someone other than the patient. Furthermore, it is important that physicians always inform patients of what to do in case of the most common doubt: whether or not they have taken their medication [42].

Promoting the use of memory tricks or other systems of reinforcement is one way that physicians can contribute to patient safety. In addition, practitioners should be especially vigilant with patients that have a pet at home. This is because people often keep their pets’ medication in the same place as their own, giving rise to some possible confusion [46]. During the consultation, physicians may enquire whether patients use a pill organiser and who is responsible for preparing it each week. 

Moreover, many patients state that they have understood the information given to them, but they still leave the clinic with unanswered questions and resort to other—sometimes questionable—sources for advice (i.e., the internet, family members, other patients). Thus, it is necessary to directly ask patients if they have any doubts, despite the potentially limited time permitted for the consultation.

Therefore, in order to address this challenge, we propose the following recommendations. (1) The participation of patients in their own safety is an element that should be included in programmes that foster self-care and shared decision-making. (2) Medication errors and adherence to the treatment regimen are key factors determining the accomplishment of therapeutic objectives; these issues also contribute to the sustainability of the health system. (3) Primary care physicians and nurses have a decisive role in achieving full patient involvement in self-care. (4) The minutes spent in the consultation help clinicians understand patients’ needs, expectations, fears, and ailments, and they can also be used to engage people in being more active, capable, and involved in their own care.

Further investigation concerning new strategies and tools to reduce patient medication errors is needed.

### 3.3. Possible Limitations

It is worth highlighting that this review is a qualitative analysis of original articles reporting quantitative research, recovered through a search algorithm. However, it still represents a systematic review of currently available research, in which we perform a narrative analysis of the articles in this area. The research topic in question—medication errors by patients—is covered in the databases we consulted (e.g., MEDLINE via PubMed) but there is little controlled vocabulary or MeSH that facilitate the development of search strategies. Indeed, free text words and other descriptors are still necessary, complicating the creation of word combinations that optimise the recovery of relevant information. Given this challenge, we used a combination of free and controlled vocabulary in order to obtain a more precise strategy. The studies reviewed are from some developed countries and regions. The number of elderly patients taking five or more medications, physicians’ information styles, or patients’ health literacy can vary in other places across the world. Elderly people are increasingly empowered in the use of electronic tools.

## 4. Conclusions

Our literature review has found that most elderly people take five or more medications a day and are treated by several physicians at once. When the number of prescriptions rises, it is more common to confuse the medications. The most common errors are related to the wrong frequency of administration, therapeutic duplication, and drug omission. In addition, polymedicated patients have a limited knowledge of prescribed medication. They are most knowledgeable about which medicines they are taking and are least knowledgeable about the precautions they should take when using their medications. Physicians must considerer this outcome and systematically introduce information about the precautions the patient must adopt to take the medication safely. 

With regard to the rate of non-adherence, this review shows that it exceeds 50% among the polymedicated elderly, and the complexity of the therapeutic regimen, the confusion surrounding generics, the lack of understanding about pharmacotherapeutic regimens, and the number of prescribers are the most frequent causes. To improve adherence and reduce errors, chronic patients use memory tricks or other systems of reinforcement. Moreover, existing studies show that PDS increases adherence and that new tools have been recently developed to improve adherence and reduce errors. Evidence also shows that elderly patients can understand written information and the presentation and format is an important factor, even in the case of polypharmacy. Previous studies have also found that a high percentage of patients have remaining doubts about their medication after their visit, with written information and the consultation length being important factors.

## Figures and Tables

**Table 1:** Main topic, country, and evidence level of the reviewed research studies.

Main Topic (*n*)	Country (*n*)	Evidence Levels * (*n*)
Adverse Drugs Events (7)	Canada (1)	
	Italy (1)	2^++^ B (2)
	United Kingdom (1)	2^+^ C (4)
	USA (2)	
	Spain (2)	
Medication Adherence and Misuse (12)	Austria (1)	2^++^ B (2)
	Canada (1)	2^+^ B (1)
	Germany (1)	2^+^ C (6)
	Spain (4)	2^−^ C (1)
	The Netherlands (1)	4 D (2)
	USA (4)	
Medication Errors (13)	Austria (1)	2^++^ B (3)
	Ireland (1)	2^+^ B (1)
	Israel (1)	2^+^ C (6)
	United Kingdom (1)	2^−^ C (2)
	USA (2)	3 D (1)
	Spain (6)	
	Sweden (1)	
Medication Management (12)	Australia (2)	1^−^ B (1)
	USA (3)	2^+^ B (1)
	Spain (6)	2^+^ C (6)
	Tokyo (1)	2^−^ C (2)
		4 D (2)
Medication Safety Information (20)	Australia (1)	4 D (6)
	Canada (2)	2^++^ B (2)
	United Kingdom (7)	2^+^ B (2)
	USA (5)	2^+^ C (5)
	Spain (5)	2^−^ C (5)
Medication Safety Tools (9)	Norway (1)	2^++^ B (1)
	USA (3)	2^+^ C (3)
	Spain (2)	2^−^ C (2)
	Taiwan (2)	3 D (2)
	United Kingdom (1)	4 D (1)
Patient Medication Knowledge (7)	Hong Kong (1)	
	Spain (2)	2^++^ B (2)
	Turkey (1)	2^+^ C (3)
	United Kingdom (1)	2^−^ C (2)
	USA (2)	

* Evidence levels and recommendation grades according to the Scottish Intercollegiate Guidelines Network (SIGN) [13]. Levels of evidence: 1^++^: High quality meta-analyses, systematic reviews of RCTs, or RCTs with a very low risk of bias. 1^+^: Well-conducted meta-analyses, systematic reviews, or RCTs with a low risk of bias. 1^−^: Meta-analyses, systematic reviews, or RCTs with a high risk of bias. 2^++^: High quality systematic reviews of case control or cohort or studies; high quality case control or cohort studies with a very low risk of confounding or bias and a high probability that the relationship is causal. 2^+^: Well-conducted case control or cohort studies with a low risk of confounding or bias and a moderate probability that the relationship is causal. 2^−^: Case control or cohort studies with a high risk of confounding or bias and a significant risk that the relationship is not causal. 3: Non-analytic studies, e.g., case reports, case series. 4: Expert opinion. Grades of recommendations: (**A**) At least one meta-analysis, systematic review, or RCT rated as 1^++^ and directly applicable to the target population; or a body of evidence consisting principally of studies rated as 1^+^, directly applicable to the target population, and demonstrating overall consistency of results. (**B**) A body of evidence including studies rated as 2^++^, directly applicable to the target population, and demonstrating overall consistency of results; or extrapolated evidence from studies rated as 1^++^ or 1^+^. (**C**) A body of evidence including studies rated as 2^+^, directly applicable to the target population and demonstrating overall consistency of results; or extrapolated evidence from studies rated as 2^++^. (**D**) Evidence level 3 or 4; or extrapolated evidence from studies rated as 2^+^.
